# Visual Performance after Bilateral Implantation of a Four-Haptic Diffractive Toric Multifocal Intraocular Lens in High Myopes

**DOI:** 10.1155/2016/5320105

**Published:** 2016-08-02

**Authors:** John S. M. Chang, Vincent K. C. Chan, Jack C. M. Ng, Antony K. P. Law

**Affiliations:** Department of Ophthalmology, Hong Kong Sanatorium and Hospital, 8/F, Li Shu Pui Block, Phase II, 2 Village Road, Happy Valley, Hong Kong

## Abstract

*Background*. The vision with diffractive toric multifocal intraocular lenses after cataract surgery in long eyes has not been studied previously.* Objectives*. To report visual performance after bilateral implantation of a diffractive toric multifocal intraocular lens in high myopes.* Methods*. Prospective, observational case series to include patients with axial length of ≥26 mm and corneal astigmatism of >1 dioptre who underwent bilateral AT LISA 909M implantation. Postoperative examinations included photopic and mesopic distance, intermediate, and near visual acuity; photopic contrast sensitivity; visual symptoms (0–5); satisfaction (1–5); and spectacle independence rate.* Results*. Twenty-eight eyes (14 patients) were included. Postoperatively, mean photopic monocular uncorrected distance, intermediate, and near visual acuities (logMAR) were 0.12 ± 0.20 (standard deviation), 0.24 ± 0.16, and 0.29 ± 0.21, respectively. Corresponding binocular values were −0.01 ± 0.14, 0.13 ± 0.12, and 0.20 ± 0.19, respectively. One eye (4%) had one-line loss in vision. Under mesopic condition, intermediate vision and near vision decreased significantly (all *P* ≤ 0.001). Contrast sensitivity at all spatial frequencies did not improve significantly under binocular condition (all *P* > 0.05). Median scores for halos, night glare, starbursts, and satisfaction were 0.50, 0.00, 0.00, and 4.25, respectively. Ten patients (71%) reported complete spectacle independence.* Conclusions*. Bilateral implantation of the intraocular lens in high myopes appeared to be safe and achieved good visual performance and high satisfaction.

## 1. Introduction

Implantation of multifocal intraocular lenses (IOL) restores vision over a range of distances and reduces spectacle dependence after cataract surgery and refractive lens exchange [1–3]. A key factor in achieving spectacle independence and patient satisfaction is precise control of the postoperative refractive error. Distance visual acuity, intermediate visual acuity, and near visual acuity of diffractive multifocal IOLs can be compromised with the presence of residual astigmatism [4]. Several approaches can be used to correct astigmatism in cataract surgery, for example, limbal relaxing incisions, bioptics, and implantation of a toric IOL. Toric IOL implantation represents the only viable option that provides predictable refractive outcomes and at the same time does not require additional surgery [5–10].

Patients with substantial corneal astigmatism and high axial myopia have limited choices of toric multifocal IOLs (TMIOLs) because a low or negative dioptric power is required. Currently, four TMIOLs are commercially available and only the AT LISA 909M (Carl Zeiss Meditec AG, Jena, Germany) provides negative dioptric power [11]. This TMIOL has been shown to be effective in restoring vision at various distances [12–14] and correcting astigmatism [12–17]. However, studies of this TMIOL included only eyes with an average axial length (AL) (range, 23.19 to 24.25 mm). In the current study, we evaluated the monocular and binocular distance, intermediate, and near visual acuities (VAs) under both photopic and mesopic conditions; monocular and binocular contrast sensitivity (CS) under photopic condition; visual symptoms; patient satisfaction; and spectacle independence in high myopes after bilateral implantation of the AT LISA 909M TMIOL.

## 2. Methods

### 2.1. Patients

This prospective, observational case series included patients who had bilateral implantation of the AT LISA 909M TMIOL after cataract surgery between May 2011 and March 2015 at the Hong Kong Sanatorium and Hospital. The inclusion criteria were an AL of 26 mm or longer [18–22], corneal astigmatism exceeding one dioptre (D), and a follow-up period of six months or more. The exclusion criteria were systemic diseases that may affect the postoperative VA (e.g., uncontrolled diabetes mellitus), capsule or zonular abnormalities that may affect postoperative IOL centration or tilt (e.g., pseudoexfoliation syndrome and Marfan's syndrome), and a history of corneal refractive surgery. The ethics committee of our hospital approved the study.

### 2.2. Intraocular Lens

The 909M is a single-piece, foldable, acrylic TMIOL with +3.75 D near addition (~+3.00 D at the spectacle plane). The overall diameter is 11 mm and the optic diameter is 6 mm. It has a four-haptic design with an aspheric, toric anterior surface and a posterior diffractive surface. The aspheric surface corrects for +0.18 *μ*m spherical aberration at 6 mm pupil (email communication with Carl Zeiss Meditec AG, 2015.). The energy distribution between the distance and near foci is asymmetrical (65% for distance focus and 35% to near focus) and independent of pupillary size [12, 14, 15].

### 2.3. Surgical Technique

The same surgeon (John S. M. Chang) performed all surgeries under topical oxybuprocaine 0.4% and intracameral lidocaine 1% or 2%. Preoperatively, the surgeon used nepafenac ophthalmic suspension 0.1% (Nevanac, Alcon Laboratories Inc., Fort Worth, TX) and tropicamide 0.5% phenylephrine hydrochloride 0.5% (Mydrin-P, Santen Pharmaceutical Co., Ltd., Osaka, Japan). The vertical meridian of the eyes was marked at the limbus under the slit lamp with the patient sitting upright. A 2.25 mm clear corneal incision was created either superiorly or temporally with a keratome. DisCoVisc ophthalmic viscosurgical device (OVD) (Alcon Laboratories Inc.) was injected into the anterior chamber and continuous curvilinear capsulorhexis was created with forceps. After hydrodissection and nucleus splitting, coaxial phacoemulsification was performed using the Infiniti Vision System (Alcon Laboratories Inc.). Irrigation and aspiration of the residual cortex and posterior capsule polishing were performed using a coaxial system. The cleared capsular bag was then filled with DisCoVisc OVD for IOL implantation. Next, the two vertical marks at the limbus were used to position the Gimbel-Mendez Fixation Ring (Mastel Precision, Rapid City, SD) and the intended TMIOL axis orientation was marked on the cornea using a coloured marker for intraoperative alignment of the TMIOL. The TMIOL was implanted into the capsular bag and then manipulated until its two linear marks were aligned with the corneal marks. The OVD was removed and the surgeon ascertained that the TMIOL remained correctly orientated before the surgery concluded.

During the postoperative period, neodymium-doped yttrium aluminium garnet (Nd:YAG) laser was performed if there was evidence of posterior capsular opacification (PCO) that affected the vision.

### 2.4. Preoperative and Postoperative Examination

A comprehensive eye examination was performed preoperatively, which included a detailed history with specific attention to the presence of dry eyes, visual distortion, and systemic diseases; other examinations included Goldmann applanation tonometry, slit-lamp biomicroscopy, and fundus examination. Corneal topography (Orbscan IIz (Bausch & Lomb, Rochester, NY) or WaveLight Oculyzer (Alcon Laboratories Inc.)) was performed in some of the patients after the current study has commenced. The IOLMaster (Carl Zeiss Meditec AG) was used to acquire all the ocular parameters (AL, corneal curvature, and anterior chamber depth) necessary for TMIOL power calculation using the manufacturer's online calculator (ZCalc, Carl Zeiss Meditec AG) [23]. All patients were shown a video that demonstrated visual symptoms (halo, night glare, and starbursts) and were informed about the possibility of permanent visual symptoms.

The postoperative measurement included noncycloplegic subjective refraction, VA, CS, and pupillary size. The monocular and binocular VA tests included measurement of the uncorrected distance VA (UDVA), corrected distance VA (CDVA), uncorrected intermediate VA (UIVA) at 67 cm, distance-corrected intermediate VA (DCIVA) at 67 cm, uncorrected near VA (UNVA) at 30 cm, and distance-corrected near VA (DCNVA) at 30 cm under photopic and mesopic conditions. The intermediate vision and near vision were measured using the SLOAN Two-Sided EDTRS format near vision chart (Precision Vision, La Salle, IL) designed for use at 40 cm. The actual VA in logarithm of the minimum angle of resolution (logMAR) at its corresponding distance was calculated by the visual angle subtended for statistical analyses [2]. The monocular and binocular distance-corrected photopic CS at spatial frequencies of 3, 6, 12, and 18 cycles/degree (cpd) were recorded using the CSV-1000E (Vector Vision, Greenville, OH). The photopic and mesopic pupillary sizes were measured using the Colvard Pupillometer (Oasys Medical Inc., San Dimas, CA). Photopic and mesopic assessments were performed at 85 and 3 candelas/m^2^, respectively.

The IOL rotation was evaluated under the slit lamp with reference to the orientation of the two linear marks located on the IOL after pupil dilation; the IOL rotation was compared to the intended orientation.

The patients completed a questionnaire regarding visual symptoms (halos, night glare, and starbursts), vision rating (distance, intermediate, and near), patient satisfaction, spectacle independence (distance, intermediate, and near), regrets about undergoing the surgery, and whether the patient would recommend the surgery to friends or relatives. The patients rated the level of visual symptoms from 0 to 5 (0, none; 1, very mild; 2, mild; 3, moderate; 4 severe; 5, very severe); vision rating from 1 to 5 (1, very blurry; 2, blurry; 3, fair; 4, clear; 5, very clear), and satisfaction from 1 to 5 (1, very dissatisfied; 2, dissatisfied; 3, neutral; 4, satisfied; 5, very satisfied).

### 2.5. Vector Analysis of Astigmatism

The Alpins method was used for vector analysis of the astigmatic results [24, 25]. The target refraction and achieved refraction were decomposed into the two principal meridian powers and then vertexed to the corneal plane with a 12 mm back vertex distance. The difference between the vertexed powers at the two principal meridians denoted the refractive astigmatism at the corneal plane. The astigmatic values were transformed into rectangular coordinates to derive the three fundamental vectors, namely, the target induced astigmatism (TIA), surgically induced astigmatism (SIA), and difference vector. These values were used to compute the following parameters to describe the accuracy of the astigmatic correction [24, 25]: the magnitude of error, which is the arithmetic difference between the magnitudes of the SIA and TIA, a positive value of which indicates an overcorrection and a negative value indicates undercorrection; the angle of error, which is the angle described by the vectors of the achieved correction (i.e., SIA) and intended correction (i.e., TIA), a positive value of which indicates that the achieved correction is counterclockwise to the intended axis and a negative value indicates the achieved correction is clockwise to the intended axis; the correction index, which is the ratio of the SIA to the TIA, of which the preferred ratio is 1, with a higher value indicating overcorrection and a lower value indicating undercorrection; and the index of success, which is the ratio of the difference vector to the TIA, of which the preferred value is 0.

### 2.6. Statistical Analysis

The statistical analyses included descriptive data for patient demographics and visual and refractive outcomes. The Kolmogorov-Smirnov test was performed to determine the normality of data. The paired *t*-test was performed to compare the preoperative and postoperative keratometry values. The paired *t*-test and Wilcoxon signed-rank test were performed to compare the postoperative photopic and mesopic VA. The paired *t*-test was performed to show binocular summations, defined as the difference between the binocular and better-eye distance-corrected VA and CS [26]. *P* < 0.05 was considered statistically significant. All statistical analyses were performed using SPSS version 16.0 (SPSS Inc., Chicago, IL).

## 3. Results

**Figure 1:**
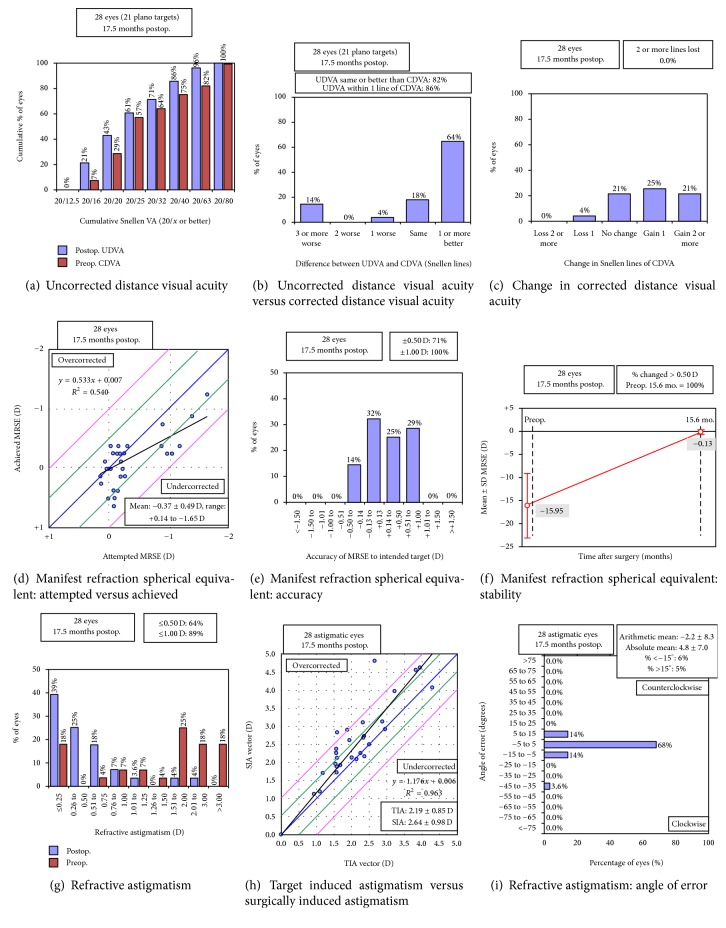
Refractive and visual outcomes (CDVA, corrected distance visual acuity; D, dioptre; MRSE, manifest refraction spherical equivalent; Postop., postoperative; Preop., preoperative; SD, standard deviation; SIA, surgically induced astigmatism; TIA, target induced astigmatism; UDVA, uncorrected distance visual acuity).

Table 1 shows the preoperative demographics and characteristics of the 28 eyes (14 patients). Corneal topography was measured on 10 patients (71%); none of them had irregular astigmatism. The mean follow-up period was 17.5 ± 10.0 months (range, 6 to 37). Intraoperative complications occurred in two eyes (7%), which include an anterior vitrectomy due to a rounded, nonextending posterior capsular tear with vitreous loss and intraoperative cracking of IOL optic requiring IOL exchange. In these cases, the IOL was implanted in the capsular bag and was well centred; there was no loss in VA. Nd:YAG laser was performed in 9 eyes (32%). No retinal detachment developed postoperatively. Data on pupillary size was available in 26 eyes (93%). The mean photopic and mesopic pupillary sizes were 3.76 ± 0.50 mm (range, 2.50 to 4.50) and 5.23 ± 0.75 mm (range, 3.00 to 6.29), respectively. Refractive and monocular visual outcomes are shown in Figure 1 and Table 2.

### 3.1. Refraction

The mean postoperative refractive error was −0.42 ± 0.48 D (range, −1.25 to 0.50) sphere and 0.59 ± 0.54 D (range, 0.00 to 2.25) cylinder with manifest refraction spherical equivalent (MRSE) of −0.13 ± 0.42 D (range, −1.25 to 0.63). Twenty-four (86%) and 27 eyes (96%) had MRSE of ±0.50 D and ±1.00 D of emmetropia, respectively. The mean error of the MRSE from the target refraction was 0.24 ± 0.34 D (range, −0.33 to 0.82). All eyes (100%) achieved MRSE of ±1.00 D from the target refraction (Figure 1). Twenty-five eyes (89%) had refractive astigmatism of 1.00 D or less (Figure 1).

### 3.2. Visual Acuity

Table 2 shows the mean monocular uncorrected and distance-corrected VAs and the numbers and percentages of eyes achieving 20/40 and 20/25 under photopic and mesopic conditions. The mean UIVA, DCIVA, UNVA, and DCNVA were significantly worse under mesopic condition than under photopic condition (*P* < 0.001 for all comparisons). One eye (4%) had VA loss from 20/20 to 20/25 (Figure 1). Both eyes (7%) of one patient had mild posterior staphyloma with a bilateral CDVA of 20/25 postoperatively.

**Figure 2:**
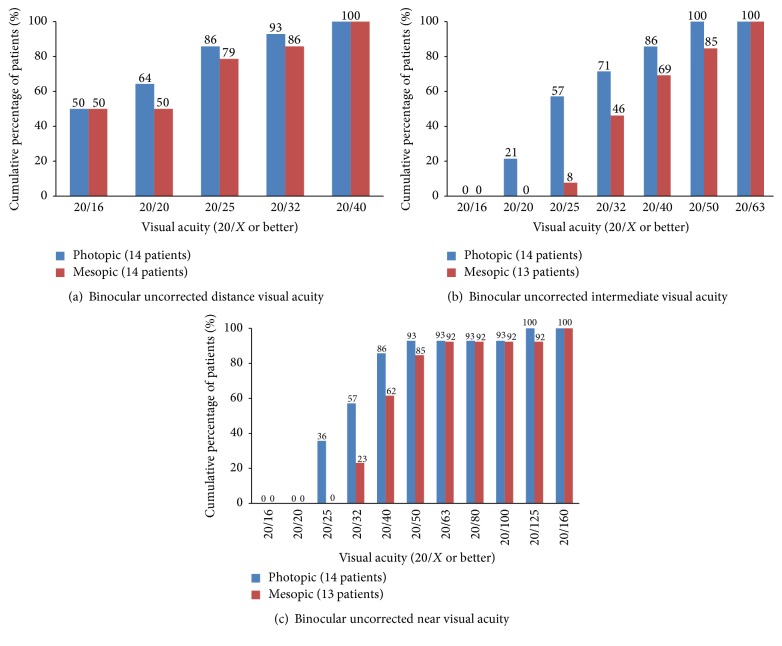
Binocular uncorrected distance, intermediate, and near visual acuity under photopic and mesopic condition at the last visit.

Table 3 shows the mean binocular uncorrected and distance-corrected VAs and the numbers and percentages of patients achieving 20/40 and 20/25 under photopic and mesopic conditions. The mean binocular UDVA, UIVA, DCIVA, UNVA, and DCNVA were significantly worse under mesopic condition than under photopic condition (*P* = 0.043, 0.001, 0.001, <0.001, and 0.001, resp.).  Figure 2 shows the cumulative percentages of binocular uncorrected distance, intermediate, and near VAs under photopic and mesopic conditions, respectively.

Under photopic condition, the mean binocular distance-corrected VAs did not significantly differ from the mean better-eye distance-corrected distance, intermediate, and near VAs (*P* = 0.336, 0.120, and 0.099, resp.). Under mesopic condition, the mean binocular distance-corrected VAs did not significantly differ from the mean better-eye CDVA (*P* = 0.165) but improved significantly compared to the mean better-eye distance-corrected intermediate and near VAs (*P* = 0.019 and 0.012, resp.).

### 3.3. Intraocular Lens Rotation

Data on IOL rotation was available in 25 eyes (89%). The mean absolute IOL rotation away from the intended orientation was 3.5 ± 5.0 degrees (range, 0 to 22). Twenty-one (84%) and 23 eyes (92%) had a rotation of 5 and 10 degrees or less, respectively.

### 3.4. Vector Analysis of Astigmatism

**Figure 3:**
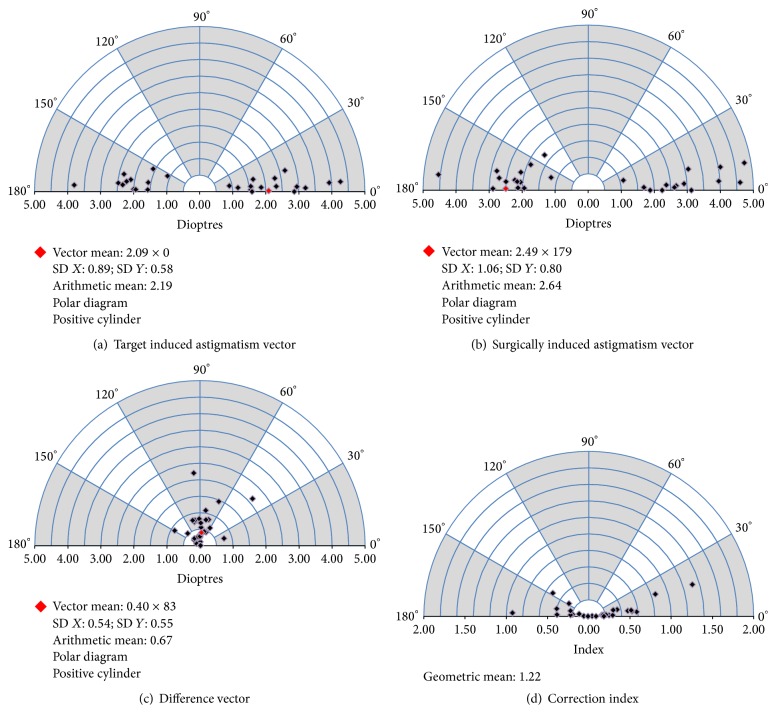
Vectorial displays (single-polar plots) for the target induced astigmatism, surgically induced astigmatism, difference vector, and correction index.

Twenty-seven eyes (96%) had preoperative with-the-rule corneal astigmatism with the axis of the steep meridian ranging from 65 to 103; one eye (4%) had oblique corneal astigmatism with steep axis at 60. The corneal astigmatism did not change significantly postoperatively (*P* = 0.314). Figures 1 and 3 and Table 4 show the vector analysis of the astigmatic results. The mean absolute angle of error was 4.78 ± 7.02 degrees (range, 0.00 to 36.69); three eyes (11%) had a large angle of error of 10.21, 13.21, and 36.69 degrees, respectively, which corresponded to misalignment of IOL axis orientation (Figure 1).

### 3.5. Contrast Sensitivity

**Figure 4:**
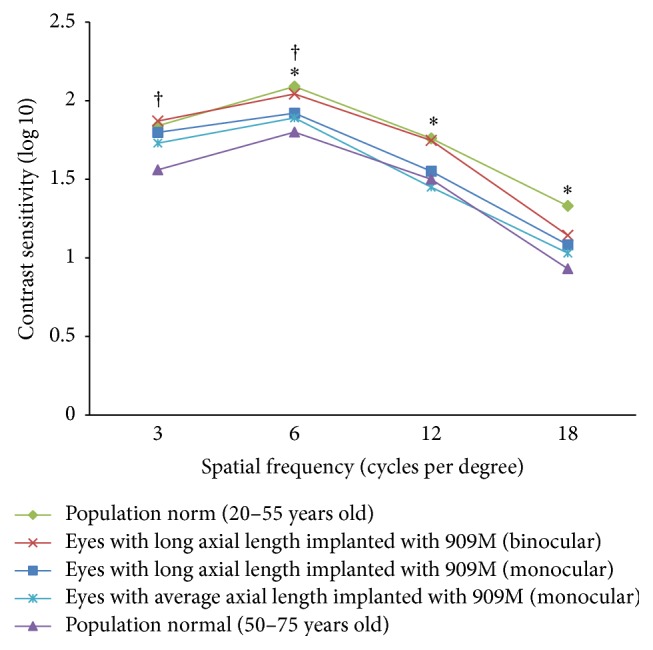
Mean monocular (*squares*) and binocular (*crosses*) contrast sensitivity at different spatial frequencies for the 909M in eyes with a long axial length in the current study and 909M in eyes with an average axial length (monocular) (*stars*, data from Visser et al. [14]) with a population norm of 20 to 55 years old (monocular) (*diamonds*, data from VectorVision [38]) and 50 to 75 years old (monocular) (*triangles*, data from Pomerance and Evans [39] and VectorVision [38]) (*∗* indicates a significant difference in mean monocular contrast sensitivity between eyes with a long axial length implanted with the 909M in the current study and the population norm of 20 to 55 years old (monocular); † indicates a significant difference in mean monocular contrast sensitivity between eyes with a long axial length implanted with the 909M in the current study and the population norm of 50 to 75 years old (monocular)).

Figure 4 shows the monocular (data available in 22 eyes) and binocular (data available in 12 patients) CS at spatial frequency of 3, 6, 12, and 18 cpd under photopic condition. The mean binocular photopic CS did not differ significantly from the mean better-eye CS at 3, 6, 12, and 18 cpd (*P* = 0.666, 0.165, 0.224, and 1.000).

### 3.6. Questionnaire

Table 5 shows the mean and median levels of visual symptoms, vision rating, and patient satisfaction. Seven (50%), five (36%), and four (29%) patients reported halos, night glare, and starbursts, respectively. Among the symptomatic patients, one (14%), 0 (0%), and 0 (0%) reported moderate symptoms (score, 3), respectively. No patients reported severe or very severe symptoms (score, >3). Fourteen (100%), nine (64%), and 13 patients (93%) rated their vision as clear (score, 4) or very clear (score, 5) at far distance, intermediate distance, and near distance, respectively. Thirteen patients (93%) were satisfied (score, 4) or very satisfied (score, 5) with the bilateral surgery; no patient was dissatisfied. Ten patients (71%) were completely spectacle independent (Table 5).

## 4. Discussion

This is the first prospective study of the visual outcomes and patient satisfaction after bilateral implantation of the AT LISA 909M TMIOL in high myopes. Previous cataract research of high myopic eyes focused primarily on monofocal IOL implantation [18, 22, 27, 28] or did not report the outcomes regarding the type of IOL used [19, 22, 29, 30]. The results showed that cataract surgery in highly myopic eyes was associated with worse VA, poorer CS, or higher risk of retinal complications compared to eyes with an average AL. Fernández-Vega et al. [31] compared the distance and near VAs and CS after implantation of the nontoric AT LISA 809M multifocal IOL (Carl Zeiss Meditec AG) between high and low-to-moderate myopic eyes and found no significant differences between the groups. Alfonso et al. [32] reported better results for distance and near VAs and CS in a group of low rather than highly myopic eyes after the implantation of the nontoric ReSTOR SN60D3 multifocal IOL (Alcon Laboratories Inc.). Ogawa et al. [21] compared the distance and near VAs and CS of Tecnis multifocal IOL (Abbott Medical Optics, Inc., Santa Ana, CA) between eyes with an AL <26 mm and ≥26 mm and found no significant differences between groups. Neither study reported monocular intermediate VA or quantified visual symptoms.

The presence of maculopathy has been associated with a poor CDVA after cataract surgery in highly myopic eyes [27, 29], while highly myopic eyes without maculopathy could achieve similar postoperative outcomes to eyes with an average AL [21, 27]. In the current study, two of the eight eyes with a postoperative CDVA worse than 20/20 had mild posterior staphyloma; none of the 24 eyes with a postoperative CDVA of 20/20 or better had posterior staphyloma. In other words, there was a higher risk of achieving poorer CDVA in eyes with maculopathy. Nevertheless, the mean CDVA of 20/19 is consistent with previous studies of the 909M in eyes with an average AL (range, 20/22 to 20/14) [12–16, 33]. Under mesopic condition, the current mean CDVA did not worsen and is possibly explained by the distance-dominant nature of the AT LISA multifocal IOLs [34] and the aspheric profile that corrects spherical aberration under dim light [3].

Regarding near vision, the 909M provided a mean monocular DCNVA of 20/35 at 30 cm in the current study, which appears to be worse than the reported value of 20/28 at 40 cm that Bellucci et al. [12] reported and other bifocal multifocal IOLs with a similar near addition, at 30 to 33 cm (range, 20/25 to 20/20) [2, 3, 31–35]. Under mesopic condition, the mean DCNVA decreased by one line from 20/35 to 20/46. The distance-dominant design of the AT LISA bifocal multifocal IOLs assumes that the patients read under normal light condition [14]. Therefore, in dim light, 35% of refracted light to near portion would be insufficient to sustain clear near vision [36], not to mention the inevitable energy loss of the diffractive optic design; however, bilateral implantation significantly improved the mesopic DCNVA to 20/38.

The current mean monocular DCIVA at 67 cm was 20/39, which was not as good as the distance and near vision but was within the reported values of other studies of the 909M (range, 20/66 to 20/23) [12–14] and 809M (range, 20/47 to 20/28) [3, 35] in eyes with an average AL at 60 to 80 cm. The mean mesopic DCIVA was 20/56 and was significantly worse than that of the photopic DCIVA because of insufficient light energy with the 909M bifocal essence [36]. The mesopic DCIVA improved insignificantly to 20/45 under binocular viewing condition.

Previous studies showed that highly myopic eyes had worse CS than other eyes under phakic [33, 37], monofocal pseudophakic [27], and multifocal pseudophakic [32] conditions. It was a general agreement that the reduced sensitivity of the postreceptoral processes [18, 37] or morphological changes in retina [18, 22, 27, 37] in highly myopic eyes may play a role.

Nevertheless, the current monocular photopic CS was comparable to two general populations across different spatial frequencies. The current results were worse than those in a young population aged between 20 and 55 years [38] at spatial frequencies of 6, 12, and 18 cpd (*P* < 0.001 for all comparisons; independent two-sample *t*-test) but better than another population aged between 50 and 75 years [38, 39] at spatial frequencies of 3 and 6 cpd (*P* < 0.001  and  =0.019, resp.; independent two-sample *t*-test) (Figure 4). The current CS also did not differ significantly from that of eyes with an average AL implanted with the 909M at all spatial frequencies (*P* > 0.05 for all comparisons; independent two-sample *t*-test) (Figure 4) [14]. Three eyes in the current study had postoperative monocular CS substantially lower (more than 40%) than the mean value of the cohort at high spatial frequencies, among which two had posterior staphyloma. Therefore, the retinal status also determined the postoperative visual quality. Highly myopic eyes still achieved good visual quality postoperatively as long as the macula was normal. A thorough preoperative examination on retinal status before cataract surgery for high myopes, especially with optical coherence tomography, is of paramount importance to manage patient expectations [33].

IOL power calculation is challenging in eyes with a long AL because IOLs of low or negative dioptric power have a different geometry from the others [20]. Undesirable hyperopic error may occur [20, 28] and the errors were greater with an increasing AL [20, 28, 40]. Inaccurate AL measurement in eyes with deep posterior staphyloma using ultrasound biometry also can result in postoperative hyperopic errors [28, 40]. In the current study, we performed optical biometry in all eyes and the IOL power was calculated using the manufacturer's calculator. Sixty-three percent and 100% of eyes achieved MRSE within ±0.50 D and ±1.00 D from the target refraction, respectively. In the two eyes diagnosed with mild staphyloma preoperatively, the errors from target refraction were 0.54 D and −0.11 D, respectively. Overall, no obvious trend toward hyperopia (mean error, 0.24 D) was observed in this group of high myopes; the manufacturer's online IOL power calculator was reliable in achieving the targeted refraction.

The use of a 2.2 mm incision minimizes the surgically induced corneal astigmatism and improves the predictability of astigmatic correction [16, 17]. The refractive astigmatism decreased from 1.83 D to 0.59 D in the current study. However, 11% of eyes had postoperative refractive astigmatism of more than 1 D because of overcorrection or IOL axis misalignment. From the vector analysis, the manufacturer's calculator overcorrects astigmatism (magnitude of error, >0.50 D) in 12 eyes (43%). Almost all eyes in the current study had with-the-rule corneal astigmatism measured by an automated keratometer. Without considering the posterior corneal astigmatism, these eyes are more prone to astigmatic overcorrection [41, 42]. In the current study, the mean absolute IOL rotation was 3.5 degrees and in most eyes (84%) the rotation was 5 degrees or less. Previous studies have shown slightly better rotational stability of the 909M than the current study, with mean rotations ranging from 1.5 to 3.1 degrees [12–14], and 93% to 96% of eyes had less than 5 degrees of rotation [12, 14]. Toric IOL rotation tended to occur in eyes with a longer AL [43, 44], which are associated with a larger capsular bag [45, 46]. The current eyes were highly myopic, which may explain the worse rotation results compared with previous studies of the 909M.

One goal of implanting TMIOLs is spectacle independence, and the postoperative uncorrected VA and rate of spectacle independence reflect patients' vision in reality. In the current study, the mean binocular UDVA, UIVA, and UNVA were 20/20, 20/27, and 20/32, respectively. This resulted in a mean patient satisfaction score of 4.39 of 5 and a rate of complete spectacle independence of 71%. Two patients (14%) had blurry or very blurry intermediate vision, among which one required spectacles for intermediate tasks and his binocular UIVA was 20/38. This implied that good postoperative binocular UIVA does not guarantee good visual quality because the bifocal design of the 909M directs minimal light energy to the intermediate portion of the multifocal IOL [36]. To enhance image brightness, a pair of spectacles with an addition of +1.25 D shifts the distance focus of the TMIOL for intermediate tasks.

Most of the current patients reported halos and night glare and only a few perceived starbursts, but no patient rated them as severe or very severe. Visser et al. [14] also found that more than half of the patients had visual symptoms after implantation of the 909M but none reported severe symptoms. This could be attributed to the soft transition of the phase zones between the main zones of the diffractive structure of the AT LISA multifocal IOLs and the adjusted phase zones for reduction of disturbing light phenomena [12, 34].

In eyes with a long AL, there is an increased risk of retinal detachment (RD) after cataract surgery [19, 47–49]. The reported rates of RD after phacoemulsification have ranged from 0% to 1.72% six months postoperatively [19, 27, 30] but reached 1.9% at two to three years postoperatively [30, 49]. In a long-term follow-up of five years, the rate increased to a range between 2.3% and 3.8% [30, 50]. Neuhann et al. [30] conducted an epidemiological study and reported that 70% of postoperative RD occurred within two years after phacoemulsification. However, no postoperative RD developed in any eyes during the mean follow-up period of 17.0 months although the current patients had a long AL (mean, 29.16 mm) and other significant independent risk factors including young age [30, 47, 48] (mean, 48.2 years) and intraoperative complications such as posterior capsular tear with subsequent anterior vitrectomy [47, 49], which occurred in one eye.

Nd:YAG capsulotomy was required in 32% of the current eyes. Previous studies of the 809M and 909M have reported rates between 3.1% and 14% six months postoperatively [3, 12, 13, 17]. A few reasons may explain the poorer current results. First, the follow-up period in the current study was longer than previous studies. Second, the current patients were younger at cataract surgery [51] than those in other studies of AT LISA multifocal IOLs, in which their patient ages ranged from 51.1 to 58.3 years. Furthermore, the plate haptic with zero-degree angulation of the AT LISA multifocal IOLs is also a risk factor for PCO [3]. Since a larger capsular bag size in highly myopic eyes may be more prone to epithelial cell migration [52], the interaction with these features requires further clarification. Although Nd:YAG capsulotomy is a controversial risk factor for postoperative RD in average eyes [30, 47, 48], one study [47] found it to be a risk factor in highly myopic eyes. Therefore, carefully monitoring remains important in the current patients.

The current study has limitations. First, most of the current patients were female and this limited the generalizability to male population. Second, the mesopic CS was not measured for a more comprehensive description of the visual function at distance. Third, it would be ideal to measure the ocular higher-order aberrations and correlate them with the contrast sensitivity and visual symptoms.

In conclusion, the current study showed that implantation of the AT LISA 909M TMIOL restored the vision of high myopes at various distances. The binocular uncorrected distance and near VAs were 20/32 or better. The visual quality at intermediate distance was not as good as that at far distance and near distance, which was reflected in the vision rating and spectacle independence. Halos and night glare were prevalent but were rated mild or moderate and did not affect patient satisfaction.

## Figures and Tables

**Table 1:** Preoperative demographics and characteristics.

Parameter	Mean ± SD	Range
Number of men (%)	3 (21)	
Age (years)	48.2 ± 6.7	35, 62
Axial length (mm)	29.16 ± 2.71	26.09, 33.90
Anterior chamber depth (mm)	3.39 ± 0.28	2.84, 3.98
Average keratometry (D)	45.13 ± 1.86	42.06, 49.36
Corneal astigmatism (D)	2.31 ± 0.86	1.13, 4.72
Corrected distance visual acuity (logarithm of the minimum angle of resolution)	0.20 ± 0.22	−0.12, 0.60
Sphere (D)	−16.87 ± 6.72	−31.00, −8.25
Cylinder (D)	1.83 ± 1.22	0.00, 4.25
Manifest refraction spherical equivalent (D)	−15.95 ± 6.94	−31.00, −7.13
IOL sphere (D)	1.93 ± 5.71	−8.0, 9.5
IOL cylinder (D)	3.34 ± 1.08	2.0, 6.0

D, dioptre; IOL, intraocular lens.

**Table 2:** Monocular visual acuity at the last visit (28 eyes).

Parameter	Mean Snellen equivalent	Mean ± SD (logMAR)	Range (logMAR)	20/40 or better, *n* (%)	20/25 or better, *n* (%)	*P* value^*∗*^
*Distance*						
Photopic UDVA	20/26	0.12 ± 0.20	−0.12, 0.54	24 (86)	17 (61)	0.379
Mesopic UDVA	20/27	0.12 ± 0.20	−0.12, 0.60	26 (93)	16 (57)	
Photopic CDVA	20/19	−0.02 ± 0.13	−0.12, 0.30	28 (100)	26 (93)	1.000
Mesopic CDVA	20/19	−0.02 ± 0.13	−0.12, 0.30	28 (100)	26 (93)	
*Intermediate*						
Photopic UIVA	20/35	0.24 ± 0.16	−0.03, 0.57	18 (64)	7 (25)	<0.001
Mesopic UIVA^†^	20/45	0.35 ± 0.14	0.05, 0.57	9 (35)	1 (4)	
Photopic DCIVA	20/39	0.29 ± 0.14	0.07, 0.57	15 (54)	4 (14)	<0.001
Mesopic DCIVA^†^	20/56	0.44 ± 0.13	0.23, 0.67	4 (15)	0 (0)	
*Near*						
Photopic UNVA	20/39	0.29 ± 0.21	0.02, 0.84	19 (68)	3 (11)	<0.001
Mesopic UNVA^†^	20/50	0.39 ± 0.19	0.12, 0.92	10 (39)	0 (0)	
Photopic DCNVA	20/35	0.24 ± 0.19	0.02, 0.74	20 (71)	7 (25)	<0.001
Mesopic DCNVA^†^	20/46	0.37 ± 0.19	0.14, 0.86	11 (42)	0 (0)	

^*∗*^Comparison between the mean photopic and mesopic values.

^†^Data on 26 eyes are available.

CDVA, corrected distance visual acuity; DCIVA, distance-corrected intermediate visual acuity; DCNVA, distance-corrected near visual acuity; logMAR, logarithm of the minimum angle of resolution; UDVA, uncorrected distance visual acuity; UIVA, uncorrected intermediate visual acuity; UNVA, uncorrected near visual acuity.

**Table 3:** Binocular visual acuity at the last visit (14 patients).

Parameter	Mean Snellen equivalent	Mean ± SD (logMAR)	Range (logMAR)	20/40 or better, *n* (%)	20/25 or better, *n* (%)	*P* value^*∗*^
*Distance*						
Photopic UDVA	20/20	−0.01 ± 0.14	−0.12, 0.30	14 (100)	12 (86)	0.043
Mesopic UDVA	20/21	0.02 ± 0.16	−0.12, 0.30	14 (100)	11 (79)	
Photopic CDVA	20/18	−0.03 ± 0.12	−0.12, 0.30	14 (100)	13 (93)	1.000
Mesopic CDVA	20/18	−0.03 ± 0.12	−0.12, 0.30	14 (100)	13 (93)	
*Intermediate*						
Photopic UIVA	20/27	0.13 ± 0.12	−0.03, 0.31	12 (86)	8 (57)	0.001
Mesopic UIVA^†^	20/36	0.26 ± 0.13	0.03, 0.47	9 (69)	1 (8)	
Photopic DCIVA	20/33	0.21 ± 0.18	−0.03, 0.53	19 (71)	5 (36)	0.001
Mesopic DCIVA^†^	20/45	0.35 ± 0.13	0.17, 0.61	6 (46)	0 (0)	
*Near*						
Photopic UNVA	20/32	0.20 ± 0.19	0.02, 0.76	12 (86)	5 (36)	<0.001
Mesopic UNVA^†^	20/40	0.30 ± 0.17	0.12, 0.80	8 (62)	0 (0)	
Photopic DCNVA	20/30	0.18 ± 0.18	0.02, 0.72	12 (86)	5 (36)	0.001
Mesopic DCNVA^†^	20/38	0.28 ± 0.18	0.12, 0.82	10 (77)	0 (0)	

^*∗*^Comparison between the mean photopic and mesopic values.

^†^Data on 13 patients are available.

CDVA, corrected distance visual acuity; DCIVA, distance-corrected intermediate visual acuity; DCNVA, distance-corrected near visual acuity; logMAR, logarithm of the minimum angle of resolution; UDVA, uncorrected distance visual acuity; UIVA, uncorrected intermediate visual acuity; UNVA, uncorrected near visual acuity.

**Table 4:** Vectorial analysis at the last visit (28 eyes).

Parameter	Arithmetic mean ± SD	Range	Vector mean	Geometric mean
Target induced astigmatism (D)	2.19 ± 0.85	0.92, 4.27	0.89 × 0.58	—
Surgically induced astigmatism (D)	2.64 ± 0.98	1.11, 4.80	1.06 × 0.80	—
Difference vector (D)	0.67 ± 0.54	0.02, 2.21	0.54 × 0.55	—
Magnitude of error (D)	0.45 ± 0.50	−0.21, 2.15	—	—
Angle of error (degrees)	−2.15 ± 8.25	−36.69, 6.79	—	—
Absolute angle of error (degrees)	4.78 ± 7.02	0.00, 36.69	—	—
Correction index	1.23 ± 0.23	0.92, 1.81	—	1.22
Index of success	0.34 ± 0.29	0.01, 1.29	—	0.28

D, dioptre.

**Table 5:** Results of questionnaire at the last visit (14 patients).

Parameter	Mean ± SD	Median	Range
*Visual symptoms* ^†^			
Halo	0.82 ± 0.99	0.50	0.0, 3.0
Night glare	0.61 ± 0.90	0.00	0.0, 2.5
Starbursts	0.43 ± 0.76	0.00	0.0, 2.0
*Vision rating* ^‡^			
Distance	4.64 ± 0.36	4.50	4.0, 5.0
Intermediate	3.57 ± 1.34	4.00	0.5, 5.0
Near	4.46 ± 0.50	4.50	3.5, 5.0
*Satisfaction* ^§^	4.39 ± 0.53	4.25	3.5, 5.0
*Number of patients (%) who regretted undergoing the surgery*	0 (0)		
*Number of patients (%) who would recommend the surgery to their friends or relatives*	13 (93)		
*Number of patients (%) who did not use spectacles for*			
Distance tasks	14 (100)		
Intermediate tasks	12 (86)		
Near tasks	11 (79)		
Any distances	10 (71)		

^†^Level of visual symptoms (0, none; 1, very mild; 2, mild; 3, moderate; 4, severe; 5, very severe).

^‡^Vision rating (1, very blurry; 2, blurry; 3, fair; 4, clear; 5, very clear).

^§^Level of satisfaction (1, very dissatisfied; 2, dissatisfied; 3, neutral; 4, satisfied; 5, very satisfied).
